# Reproducibility and Relative Validity of a Food Frequency Questionnaire Developed for Adults in Taizhou, China

**DOI:** 10.1371/journal.pone.0048341

**Published:** 2012-11-06

**Authors:** Maoqiang Zhuang, Ziyu Yuan, Lanfang Lin, Bin Hu, Xiaofeng Wang, Yajun Yang, Xingdong Chen, Li Jin, Ming Lu, Weimin Ye

**Affiliations:** 1 Clinical Epidemiology Unit, Qilu Hospital of Shandong University, Jinan, Shandong, People’s Republic of China; 2 CMC Institute of Health Sciences, Taizhou, Jiangsu, People’s Republic of China; 3 MOE Key Laboratory of Contemporary Anthropology, School of Life Sciences and Institutes of Biomedical Sciences, Fudan University, Shanghai, People’s Republic of China; 4 Department of Medical Epidemiology and Biostatistics, Karolinska Institutet, Stockholm, Sweden; Sookmyung Women's University, Republic of Korea

## Abstract

**Objective:**

To evaluate the reproducibility and validity of a food frequency questionnaire (FFQ) developed to investigate the relationship between dietary factors and diseases in the adult Chinese population in East China.

**Methods:**

A total of 78 males and 129 females aged 30–75 years completed four inconsecutive 24-hour dietary recalls (24-HRs, served as a reference method) and two FFQs (FFQ1 and FFQ2) over a nine-month interval. The reproducibility of the FFQ was estimated with correlation coefficients, cross-classification, and weighted kappa statistic. The validity was assessed by comparing the data obtained from FFQ and 24-HRs.

**Results:**

The median nutrient intakes assessed with FFQs were higher than the average of four 24-HRs. For the food groups, Spearman, Pearson, and intraclass correlation coefficients between FFQ1 and FFQ2 ranged from 0.23 to 0.61, 0.27 to 0.64, and 0.26 to 0.65, respectively. For total energy and nutrient intakes, the corresponding coefficients ranged from 0.25 to 0.61, 0.28 to 0.64, and 0.28 to 0.62, respectively. The correlations between FFQ1 and FFQ2 for most nutrients decreased after adjustment with total energy intake. More than 70% of the subjects were classified into the same and adjacent categories by both FFQs. For food groups, the crude, energy-adjusted, and de-attenuated Spearman correlation coefficients between FFQ2 and the 24-HRs ranged from 0.17 to 0.59, 0.10 to 0.57, and 0.11 to 0.64, respectively. For total energy and nutrient intakes, the corresponding coefficients ranged from 0.20 to 0.58, 0.08 to 0.54, and 0.09 to 0.56, respectively. More than 67% of the subjects were classified into the same and adjacent categories by both instruments. Both weighted kappa statistic and Bland-Altman Plots showed reasonably acceptable agreement between the FFQ2 and 24-HRs.

**Conclusion:**

The FFQ developed for adults in the Taizhou area is reasonably reliable and valid for assessment of most food and nutrient intakes.

## Introduction

The global public health burden of chronic diseases, particularly cancer and cardiovascular disease, is growing rapidly. Some of these diseases are designated as preventable with lifestyle changes of people including dietary factors [1]. Previous studies have shown that various nutrients are associated with development of cancers although controversy remains [2], [3]. To determine the relationship between nutrients and human diseases, it is important to accurately assess the food and nutrient intakes. However, accurate assessment of food intakes of free-living persons is difficult and labor-intensive and requires culturally sensitive and valid measurement instrument. Weighted food record is one of the most accurate methods, however, it is time consuming and generally suitable only for individuals or small groups of cooperative volunteers [4]. In addition, the main limitation of this method is that the collected data only represent the short-term intake of individuals. For long-term dietary intakes of months or years, the most practical and efficient method is food frequency questionnaire (FFQ) because of its ease of administration, low cost, and ability to rank individuals according to dietary intake [5]. In the present Taizhou longitudinal cohort study (TZL), we developed a new FFQ to estimate the nutrient and food group intakes of people in the Taizhou area. The TZL, initiated in 2007 in Jiangsu of China, was a population-based open-ended prospective cohort study with major objective to investigate risk factors of chronic non-communicable disease, especially cancer. The design and baseline characteristics of this study have been described previously [6]. Because dietary habit varies greatly due to the ethnic, social, and cultural backgrounds of participants, the measurement errors can adversely affect the results for the association between diet and diseases [7]. Therefore, dietary assessment of nutrients and food groups with FFQ needs to be validated.

Currently, there is no gold standard for the validation of dietary intake. The basic requirement for validation is that the errors of reference method are independent of test method. The major sources of errors in FFQs include memory, interpretation of questions, perception of portion sizes, and the restricted food list. Diet records have the least correlated errors with FFQs [4]. But diet records bring subjects great burden, decrease the response rate, and even may change subjects’ diet. Collection of multiple 24-hour dietary recalls (24-HRs) is then widely considered as an alternative method to diet records. A critical review regarding validation of FFQs has shown that FFQs are validated against repeated 24-HRs in 75% of studies [8].

In this study, the nutrient and food group intakes assessed with FFQ were comparatively analyzed with the data obtained from four 24-HRs. The overall goal of this study was to evaluate the relative validity and reproducibility of the FFQ we developed.

## Subjects and Methods

### Ethics Statement

This study was approved by the Ethics Committee of the College of Life Sciences, Fudan University, Shanghai, China. All participants gave their written informed consent prior to participation.

### Study Population

The subjects in the present study were recruited with multi-stage stratified random sampling method. We first randomly selected five towns and then one village or community from each town (a total of 4 rural and 1 urban) based on the geographical and economic conditions. We then randomly selected 350 age- and gender-stratified subjects, aged 30–75 years, from the five villages and communities (70 subjects each village or community). Subjects were proportionately distributed across age groups and genders to generalize the results to all age groups and genders [9]. The inclusion criteria were current resident in Taizhou area for longer than 5 years, free-living people without serious diseases requiring a special diet, and not on a weight reduction diet. Of the 350 subjects, 251 subjects agreed to participate in this study (response rate: 72%) and others were excluded because of refusal, out of the area during investigation period, poor health, or other reasons.

We also collected the body weight, height, education level, smoking status (current smokers or ex-smokers), alcohol drinking status (drinkers or non-drinkers), and systolic and diastolic blood pressures of participants. Current smokers were defined as those who reported smoking at the time of interview and had a smoking history for more than 1 year with at least one cigarette per day. Ex-smokers were defined as those who smoked for more than 1 year with at least one cigarette per day, but did not smoke during the 6-month period prior to the review. None-smokers were defined as those who never smoked or smoked but did not meet the criteria of current or ex-smoker. Alcohol drinkers were defined as those who reported drinking at the time of interview and had a drinking history for more than 1 year with at least three times per week. Non-drinkers were defined as those who have never drunk or did not meet the criteria of drinkers.

### Study Design

**Figure 1:**
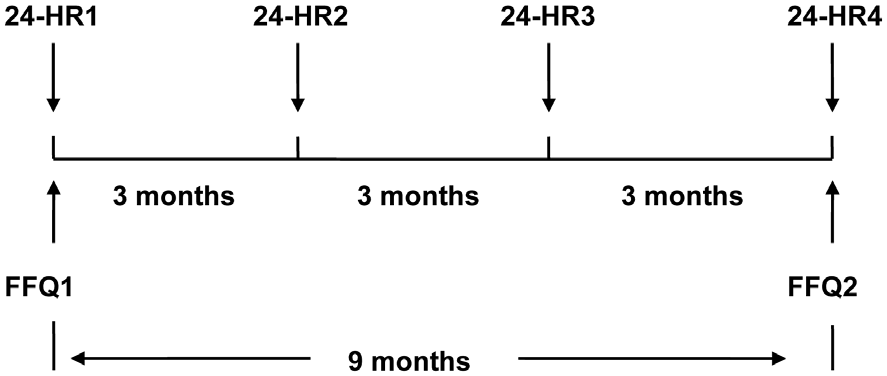
Design of the reproducibility and validation study. FFQ1 was administered during the first 24-HR and FFQ2 was administered during the last 24-HR. The four 24-HRs were administered at intervals of three months.

The study started from March 2011 and lasted for the subsequent nine months. During the study period, four inconsecutive 24-HRs were collected from each participant at intervals of three months. The first FFQ (FFQ1) was administered during the first 24-HR and the second FFQ (FFQ2) was administered in December 2011 during the last 24-HR. The study design is shown in Figure 1.

**Table 1:** Characteristics of participants in the validation study.^a^

Variables	number	%
GenderFemaleMale	12978	62.337.7
Marriage statusMarriedDivorced or widowed	19116	92.37.7
EducationJunior high school and belowSenior high school and above	17925	87.812.2
Type of JobFarmersBlue collar workersOther	796459	39.131.729.2
Smoking statusCurrent smokerEx-smokerNever smoker	564145	27.32.070.7
Alcohol drinking statusYesNo	42165	20.379.7
Income (RMB)^b^<35,000≥35,000	99105	48.551.5
	mean	SD
Age (years)	51.5	10.5
BMI (Kg/m^2^)	24.9	3.8
SBP (mmHg)	126.6	15.0
DBP (mmHg)	82.2	8.6
FPG (mmol/L)	5.4	2.0
TC (mmol/L)	4.7	1.0
TG (mmol/L)	1.5	0.9

BMI, body mass index; SBP, systolic blood pressure; DBP, diastolic blood pressure; FPG, fasting plasma glucose; TC, total cholesterol; TG, triglyceride; SD, standard deviation.

a data were from FFQ1.

b family’s annual income.

### Food Frequency Questionnaire

The FFQ was developed based on the method proposed by Willett, which included questions about average consumption and frequency during past year [4]. The food items were first selected from the most frequently consumed items listed in the National Health and Dietary Survey in China [10] and then some unlisted, commonly consumed foods in the local area obtained from pilot test were added to reflect the local dietary habits. In the end, the FFQ includes 86 food items and seven open questions. The food items were classified into 11 groups including cereal, pickled vegetables, egg, meat, milk, fish and shellfish, snack and nut, bean, vegetable, fruit, and cooking oil.

Considering that different recipes might be used for food preparation, the FFQ was developed based on food items rather than dishes. For each food item, participants were asked how frequently (daily, weekly, monthly, yearly or never) they consumed the food. The average amount consumed each time was asked in “liang”, a common unit of weight in China (1 “liang” = 50 g). We prepared a book containing colored photographs of all food items at different portion sizes to improve the accuracy of amount estimation. For seasonal vegetables and fruits, participants were asked to recall how often they ate these foods during the season.

**Table 2:** Mean and median intakes per day estimated from FFQs and 24-HRs.

variables	24-HRs		FFQ1		FFQ2	
	median	mean	median	mean	median	mean
Food groups						
Cereal (g)	562.5	596.3	526.0	596.2	421.1	458.0 ^bd^
Pickled vegetable (g)	22.5	28.8	29.2	35.6 b	25.3	30.0 d
Egg (g)	20.6	28.5	15.7	23.0 a	15.7	24.4
Meat (g)	60.4	71.5	31.9	50.6 b	31.3	45.1 b
Fish and shellfish (g)	37.5	46.4	23.3	40.2	24.1	38.2 a
Milk (g)	62.5	85.5	57.1	100.4 a	66. 7	99.0 b
Snack and nut (g)	15.6	19.1	6.6	15.2	7.3	16.7
Bean (g)	50.0	60.5	47.3	65.6	49.8	61.3
Vegetable (g)	206.3	216.5	339.8	359.2 b	303.4	336.8 b
Fruit (g)	50.0	71.0	127.0	163.8 b	98.7	125.2 ^bd^
Cooking oil (g)	32.5	34.1	38.9	40.2 b	41. 7	43.6 ^bc^
Nutrients						
Energy (Kcal)	1258.3	1365.1	1520.9	1632.7 b	1348.6	1482.2^bd^
Protein (g)	52.4	57.3	57.3	64.3 b	51.5	59.0 d
Fat (g)	47.4	49.3	51.2	55.7 b	53.4	57.6 b
Fiber (g)	8.9	10.1	15.6	16.2 b	12.7	14.8 ^bd^
Carbohydrate(g)	146.6	156.8	188.2	200.3 b	154.4	163.9 d
Vitamin A (µg)	308.2	371.4	568.6	613.1 b	536.6	572.9 ^bc^
Carotene (µg)	1372.0	1486.7	2949.1	3137.3 b	2774.1	2877.1^bc^
Thiamin (mg)	0.5	0.6	0.7	0.8 b	0.6	0.7 ^bd^
Riboflavin (mg)	0.6	0.7	0.9	1.0 b	0.8	0.9 ^bd^
Niacin (mg)	12.2	13.5	15.7	16.6 b	13.6	15.3 ^bd^
Vitamin E (mg)	25.7	26.6	29.7	31.5 b	30.7	32.8 b
Sodium (mg)	1204.8	1373.0	1525.9	1763.1 b	1339.0	1471.3^d^
Calcium (mg)	353.7	391.4	538.6	576.0 b	504.6	541.4 ^bc^
Iron (mg)	16.7	17.8	21.4	23.0 b	19.4	20.9 ^bd^
Vitamin C (mg)	48.3	51.9	95.8	103.7 b	89.7	93.8 ^bd^
Cholesterol(mg)	198.9	237.9	173.4	221.3	173.3	229.3

P values were derived from Wilcoxon sign rank test.

a P<0.05,

b P<0.01 (FFQ1 *vs.* 24-HR or FFQ2 *vs.* 24-HR).

c P<0.05,

d P<0.01 (FFQ1 *vs.* FFQ2).

### 24-hour Dietary Recall

Four non-consecutive 24-HRs were carried out at 3-month interval during the study period, which covered one weekend day and three weekdays. Each participant was asked to provide the name and amount of all foods consumed during the previous 24 hours. If the previous day was a special day due to feast or travels, et al., the food consumption of the day before the 24 hours was recorded or another day was chosen to interview the participant by telephone. Subjects were not informed of the survey until the evening before the interview. The amounts of different food items that were mixed in one dish were recorded respectively. The recalled food items were assigned to the corresponding food groups as defined by the FFQ. Trained interviewers administered the FFQs and 24-HRs by face-to-face interviews. All records were checked immediately and ambiguities were resolved with the subjects. Each participant had his or her own interviewer through the whole study period.

### Data Cleaning

Participants who did not satisfactorily complete the FFQs or missed more than one out of the four 24-HRs (n = 42) were excluded from the analyses. Subjects with implausible energy intakes (<500 Kcal or>5000 Kcal) were also excluded as described by previous studies. Extreme values were examined with scatter plots, which were generated for the mean nutrient values of the 24-HRs by plotting against the mean of FFQ1 and FFQ2 [11]. A decision about whether or not to exclude the record from analyses was made according to the original FFQs and/or 24-HRs. A total of 44 subjects were excluded from the analyses.

**Table 3:** Correlation coefficients of FFQ1 and FFQ2.

variables	Spearman		Pearson		ICC	
	R^a^	R^b^	R c	R^d^	R^c^	R^d^
Food groups						
Cereal (g)	0.53	0.50	0.50	0.53	0.42	0.45
Pickled vegetable (g)	0.23	0.23	0.28	0.19	0.30	0.28
Egg (g)	0.44	0.51	0.42	0.46	0.44	0.41
Meat (g)	0.60	0.39	0.58	0.40	0.58	0.45
Fish and shellfish (g)	0.56	0.35	0.52	0.33	0.52	0.43
Milk (g)	0.51	0.39	0.44	0.42	0.50	0.52
Snack and nut (g)	0.52	0.53	0.53	0.51	0.50	0.45
Bean (g)	0.56	0.52	0.53	0.53	0.53	0.48
Vegetable (g)	0.26	0.10	0.28	0.14	0.28	0.18
Fruit (g)	0.61	0.54	0.64	0.57	0.65	0.64
Cooking oil (g)	0.34	0.25	0.27	0.26	0.26	0.29
Nutrients						
Energy (Kcal)	0.60	−	0.59	−	0.57	−
Protein (g)	0.61	0.41	0.62	0.42	0.61	0.42
Fat (g)	0.42	0.26	0.42	0.29	0.42	0.29
Fiber (g)	0.51	0.37	0.51	0.37	0.51	0.36
Carbohydrate (g)	0.53	0.54	0.50	0.51	0.45	0.37
Vitamin A (µg)	0.35	0.17	0.34	0.17	0.34	0.23
Carotene (µg)	0.28	0.15	0.28	0.16	0.28	0.23
Thiamin (mg)	0.58	0.41	0.59	0.40	0.57	0.33
Riboflavin (mg)	0.51	0.35	0.53	0.31	0.51	0.30
Niacin (mg)	0.55	0.39	0.54	0.37	0.53	0.30
Vitamin E (mg)	0.44	0.28	0.37	0.30	0.37	0.30
Sodium (mg)	0.25	0.14	0.29	0.13	0.28	0.20
Calcium (mg)	0.42	0.14	0.43	0.24	0.43	0.27
Iron (mg)	0.59	0.26	0.56	0.27	0.55	0.27
Vitamin C (mg)	0.34	0.17	0.33	0.20	0.32	0.24
Cholesterol (mg)	0.56	0.63	0.64	0.50	0.62	0.56

a crude correlation coefficients.

b data were energy-adjusted.

c data were log-transformed.

d data were log-transformed and energy-adjusted.

**Table 4:** Misclassification and weighted kappa between FFQ1 and FFQ2.

variables	FFQ1 vs. FFQ2			Weighted kappa
	Same (%)	Adjacent (%)	Extreme (%)	
Food groups				
Cereal (g)	44.9	34.8	1.9	0.38
Pickled vegetable (g)	34.8	36.7	11.1	0.16
Egg (g)	46.4	29.5	5.8	0.35
Meat (g)	45.9	41.1	2.4	0.44
Fish and shellfish (g)	41.1	43.5	3.4	0.38
Milk (g)	65.7	20.3	5.8	0.50
Snack and nut (g)	42.5	35.3	6.3	0.33
Bean (g)	43.0	41.1	3.9	0.39
Vegetable (g)	30.9	39.1	5.3	0.16
Fruit (g)	45.9	39.1	2.4	0.43
Oil (g)	43.0	37.7	6.8	0.32
Nutrients				
Energy (Kcal)	48.3	34.3	1.5	0.44
Protein (g)	44.9	41.1	2.4	0.43
Fat (g)	40.6	38.2	6.3	0.30
Fiber (g)	40.1	41.1	1.9	0.35
Carbohydrate (g)	41.6	42.0	3.4	0.37
Vitamin A (µg)	34.3	43.0	6.3	0.24
Carotene (µg)	36.2	37.7	9.7	0.20
Thiamin (mg)	49.3	32.9	1.0	0.44
Riboflavin (mg)	41.2	37.2	2.9	0.34
Niacin (mg)	42.5	40.6	2.9	0.38
Vitamin E (mg)	39.6	39.1	5.3	0.30
Sodium (mg)	36.7	36.2	10.1	0.20
Calcium (mg)	34.3	42.0	4.4	0.25
Iron (mg)	47.8	37.7	1.9	0.45
Vitamin C (mg)	34.8	39.6	5.8	0.23
Cholesterol (mg)	43.0	37.2	2.4	0.37

### Statistical Analysis

Daily intakes of each food item were determined based on the average consumption frequency and the amount of each food item. Nutrient intake for each food item was calculated as daily intake of each food item multiplied by nutrient per 100 gram. The major nutrient composition of foods can be found in the Chinese Food Composition Tables [12]. Log-transformation was applied to improve the normality of the distribution of the food group and nutrient intakes. Validity of the FFQ was evaluated by comparing the average of four 24-HRs with data of FFQ2. Reproducibility was estimated by comparing the intakes from FFQ1 and FFQ2.

Mean and median were calculated for both FFQs and 24-HRs. Significances of the differences for intakes of food groups and nutrients between FFQ1 and FFQ2, and between FFQs and the average of four 24-HRs, were determined with Wilcoxon signed-rank test. Spearman correlation coefficients were calculated with unadjusted nutrient data, while Pearson correlation coefficients were calculated based on the adjusted data (log-transformation, energy-adjustment and de-attenuation). Energy-adjusted nutrient intakes were obtained with the regression residual method, with nutrient intakes as the dependent variable and total energy intake as the independent variable [13]. Residuals were added to the expected nutrient value for the mean energy intake of the sample. De-attenuated correlation coefficients were calculated to adjust for within-person variation [14]. The formula is: 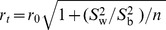, where 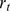 is the true correlation, 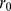 is the observed correlation, 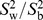 is the ratio of within- and between-person variances, and 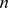 is the number of 24-HR.

The ability of the FFQ to rank dietary intakes of individuals in the population was also calculated by comparing with the mean of the recalls. Study subjects were classified into quartiles based on the crude food group and nutrient intakes from FFQ and 24-HRs. The degree of misclassification was estimated by examining the proportion of subjects classified by the reference method that fell into the same, adjacent, or extreme quartile when classified by the FFQ. Misclassification into the extreme quartile comprises both misclassifications from the first to the fourth quartile, and vice versa, from the fourth to the first quartile. Weighted kappa statistic and intraclass correlation coefficient (ICC) were also calculated [15], [16].

Bland-Altman method that plots the individual differences between two methods against the mean of the methods gives a visual comparison of assessment [17]. Therefore, the average differences between FFQs and four 24-HRs were plotted against the mean of average FFQs and 24-HRs. When Bland Altman plots showed a tendency for the differences to increase as the magnitude of the measurement increased, the data was then log-transformed and re-plotted. All statistical analyses were performed with SAS 9.1 (SAS Institute Inc., Cary, N.C.). A value of *P*<0.05 was considered to be statistically significant.

**Table 5:** Spearman correlation coefficients of food groups and nutrients estimated from FFQs and 24-HRs.

variables	FFQ1 *vs.* 24-HRs			FFQ2 *vs.* 24-HRs			Mean a *vs.* 24-HRs		
	Crude	Energy adjusted	De-attenuated^b^	Crude	Energy adjusted	De-attenuated^b^	Crude	Energy adjusted	De-attenuated^b^
Food groups									
Cereal (g)	0.58	0.56	0.58	0.54	0.57	0.59	0.65	0.66	0.68
Pickled vegetable (g)	0.16	0.23	0.26	0.17	0.10	0.11	0.25	0.23	0.26
Egg (g)	0.26	0.45	0.49	0.19	0.31	0.34	0.25	0.42	0.46
Meat (g)	0.53	0.32	0.36	0.44	0.20	0.22	0.56	0.31	0.35
Fish and shellfish (g)	0.39	0.33	0.36	0.43	0.19	0.21	0.47	0.31	0.34
Milk (g)	0.21	0.34	0.37	0.24	0.32	0.35	0.30	0.42	0.46
Snack and nut (g)	0.49	0.49	0.52	0.46	0.49	0.52	0.52	0.52	0.55
Bean (g)	0.45	0.38	0.41	0.42	0.34	0.37	0.48	0.42	0.46
Vegetable (g)	0.22	0.10	0.11	0.30	0.14	0.16	0.32	0.16	0.18
Fruit (g)	0.59	0.54	0.66	0.59	0.52	0.64	0.68	0.63	0.77
Oil (g)	0.09	0.03	0.03	0.20	0.12	0.13	0.18	0.10	0.11
Nutrients									
Energy (Kcal)	0.59	−	−	0.56	−	−	0.64	−	−
Protein (g)	0.58	0.35	0.38	0.58	0.22	0.23	0.63	0.34	0.36
Fat (g)	0.48	0.27	0.29	0.45	0.29	0.31	0.55	0.36	0.38
Fiber (g)	0.20	0.05	0.05	0.30	0.17	0.19	0.29	0.12	0.13
Carbohydrate (g)	0.42	0.57	0.59	0.42	0.54	0.56	0.49	0.62	0.64
Vitamin A (µg)	0.29	0.16	0.18	0.24	0.09	0.10	0.33	0.16	0.18
Carotene (µg)	0.24	0.12	0.14	0.20	0.11	0.12	0.28	0.14	0.16
Thiamin (mg)	0.40	0.30	0.33	0.45	0.29	0.31	0.47	0.35	0.38
Riboflavin (mg)	0.47	0.17	0.19	0.46	0.19	0.21	0.51	0.22	0.24
Niacin (mg)	0.51	0.29	0.32	0.55	0.18	0.20	0.59	0.28	0.30
Vitamin E (mg)	0.39	0.25	0.27	0.39	0.28	0.30	0.47	0.33	0.35
Sodium (mg)	0.26	0.28	0.31	0.27	0.20	0.22	0.34	0.32	0.35
Calcium (mg)	0.44	0.13	0.14	0.38	0.14	0.15	0.49	0.18	0.20
Iron (mg)	0.55	0.18	0.19	0.53	0.08	0.09	0.59	0.17	0.18
Vitamin C (mg)	0.25	0.16	0.18	0.28	0.14	0.15	0.32	0.20	0.22
Cholesterol (mg)	0.44	0.37	0.40	0.43	0.38	0.41	0.48	0.41	0.45

a mean of FFQ1 and FFQ2.

b data were energy-adjusted.

**Table 6:** Pearson correlation coefficients of food groups and nutrients estimated from FFQs and 24-HRs.^a^

Variables	FFQ1 *vs.* 24-HRs			FFQ2 *vs.* 24-HRs			Mean b *vs.* 24-HRs		
	Crude	Energy adjusted	De-attenuated c	Crude	Energy adjusted	De-attenuated c	Crude	Energy adjusted	De-attenuated c
Food groups									
Cereal (g)	0.55	0.56	0.58	0.58	0.59	0.61	0.65	0.65	0.67
Pickled vegetable (g)	0.13	0.26	0.29	0.14	0.13	0.15	0.21	0.28	0.32
Egg (g)	0.26	0.37	0.40	0.15	0.22	0.24	0.20	0.31	0.34
Meat (g)	0.49	0.29	0.32	0.44	0.22	0.24	0.53	0.32	0.36
Fish and shellfish (g)	0.36	0.27	0.30	0.44	0.31	0.34	0.45	0.33	0.37
Milk (g)	0.30	0.38	0.42	0.26	0.31	0.35	0.26	0.39	0.43
Snack and nut (g)	0.39	0.56	0.58	0.38	0.54	0.56	0.38	0.63	0.66
Bean (g)	0.47	0.36	0.39	0.45	0.41	0.45	0.51	0.45	0.49
Vegetable (g)	0.16	0.13	0.14	0.28	0.14	0.16	0.28	0.17	0.19
Fruit (g)	0.56	0.50	0.62	0.65	0.47	0.58	0.71	0.55	0.68
Cooking oil (g)	0.08	0.08	0.09	0.19	0.15	0.16	0.16	0.14	0.15
Nutrients									
Energy (Kcal)	0.63	−	−	0.61	−	−	0.69	−	−
Protein (g)	0.62	0.33	0.35	0.61	0.31	0.33	0.67	0.38	0.40
Fat (g)	0.54	0.30	0.32	0.50	0.33	0.35	0.61	0.41	0.44
Fiber (g)	0.22	0.03	0.03	0.31	0.14	0.15	0.29	0.09	0.10
Carbohydrate (g)	0.44	0.51	0.53	0.44	0.51	0.53	0.51	0.59	0.61
Vitamin A (µg)	0.29	0.15	0.17	0.29	0.11	0.12	0.35	0.17	0.19
Carotene (µg)	0.21	0.13	0.15	0.21	0.12	0.14	0.28	0.17	0.19
Thiamin (mg)	0.45	0.26	0.28	0.48	0.35	0.38	0.51	0.36	0.39
Riboflavin (mg)	0.51	0.19	0.21	0.52	0.17	0.19	0.58	0.21	0.23
Niacin (mg)	0.55	0.18	0.20	0.55	0.17	0.19	0.61	0.21	0.23
Vitamin E (mg)	0.39	0.30	0.32	0.43	0.33	0.35	0.49	0.39	0.41
Sodium (mg)	0.29	0.32	0.35	0.32	0.23	0.25	0.38	0.37	0.41
Calcium (mg)	0.41	0.19	0.21	0.41	0.19	0.21	0.47	0.24	0.26
Iron (mg)	0.55	0.20	0.22	0.55	0.12	0.13	0.61	0.21	0.23
Vitamin C (mg)	0.22	0.19	0.21	0.26	0.15	0.17	0.30	0.21	0.23
Cholesterol (mg)	0.45	0.36	0.39	0.50	0.38	0.41	0.53	0.41	0.45

a data were log-transformed.

b mean of FFQ1 and FFQ2.

c data were log-transformed and energy-adjusted.

## Results

Of the 207 participants eligible for analysis, 62.3% were females; the mean age was 51.5±10.5 years; the mean BMI was 24.9±3.8 kg/m^2^; and 87.8% had education of junior high school or below. The proportion of current smoker and drinker was 27.3% and 20.3%, respectively. More than 50% of the participants had an income ≥35,000 RMB per year (Table 1).

The median and mean intakes of total energy, nutrients, and food groups estimated from FFQs, the average of the four 24-HRs, and the results from Wilcoxon signed-rank test are presented in Table 2. Wilcoxon signed-rank test showed that the intakes of almost all nutrients and food groups obtained from FFQs were statistically significantly different from the intakes obtained from 24-HRs. The median intakes for almost all nutrients assessed with FFQ2 were lower or equal to the values obtained from FFQ1, except for fat, vitamin E, cholesterol. All the nutrients were overestimated by FFQs compared to the intakes derived from 24-HRs, except for cholesterol (FFQ1 and FFQ2). No significant trends were observed for the food groups between FFQ1 and FFQ2, or between FFQs and 24-HRs.

**Table 7:** Misclassification and weighted kappa between FFQs and 24-HRs.

Variables	FFQ1 *vs.* 24-HRs				FFQ2 *vs.* 24-HRs				Mean a *vs.* 24-HRs			
	Same (%)	Adjacent (%)	Extreme (%)	Weighted kappa	Same (%)	Adjacent (%)	Extreme (%)	Weighted kappa	Same (%)	Adjacent (%)	Extreme (%)	Weighted kappa
Food groups												
Cereal (g)	43.0	40.6	1.0	0.40	40.6	42.0	3.4	0.36	4.3	39.1	2.4	0.45
Pickled vegetable (g)	30.0	34.8	9.2	0.10	29.5	40.6	10.6	0.12	32.4	40.6	9.7	0.18
Egg (g)	31.4	34.3	9.7	0.13	37.2	31.4	9.2	0.22	34.8	36.7	7.7	0.21
Meat (g)	43.0	40.6	2.9	0.40	44.9	35.3	3.4	0.38	50.2	34.3	3.9	0.46
Fish and shellfish (g)	31.4	43.0	4.8	0.25	41.6	38.2	7.3	0.35	40.1	37.2	5.3	0.33
Milk (g)	60.4	17.9	5.8	0.26	61.4	18.4	6.3	0.28	54.1	20.8	6.3	0.28
Snack and nut (g)	39.6	25.1	11.2	0.17	36.2	31.4	11.1	0.17	35.3	30.9	11.6	0.21
Bean (g)	39.1	41.1	4.8	0.32	35.3	43.0	5.3	0.27	42.0	38.7	3.4	0.36
Vegetable (g)	34.3	32.9	7.3	0.15	33.8	38.2	7.7	0.18	33.3	39.6	7.3	0.19
Fruit (g)	33.8	35.8	9.2	0.23	37.2	33.8	7.3	0.28	38.2	31.9	6.3	0.29
Oil (g)	28.5	38.7	10.6	0.07	39.6	32.4	8.2	0.20	33.3	39.1	7.3	0.16
Nutrients												
Energy (Kcal)	42.5	39.6	1.9	0.38	40.6	42.0	2.4	0.37	47.3	37.7	1.9	0.44
Protein (mg)	44.0	41.1	1.5	0.42	44.0	38.2	1.5	0.40	41.1	43.5	1.0	0.40
Fat (mg)	36.7	44.0	2.4	0.32	36.7	39.1	4.4	0.26	41.6	39.6	1.9	0.37
Fiber (mg)	26.6	41.1	9.7	0.07	34.3	39.6	7.7	0.20	31.9	40.6	7.7	0.17
Carbohydrate (mg)	38.7	34.3	2.4	0.27	32.4	42.0	4.4	0.22	41.1	36.2	1.5	0.33
Vitamin A (µg)	33.8	39.1	6.3	0.20	32.4	34.3	8.2	0.13	33.3	39.6	5.8	0.20
Carotene (µg)	35.8	36.7	6.8	0.21	30.9	37.2	9.2	0.12	32.4	41.1	6.3	0.20
Thiamin (mg)	38.2	39.1	5.3	0.28	37.7	38.2	3.4	0.28	41.6	36.7	3.9	0.33
Riboflavin (mg)	37.7	39.1	3.4	0.29	38.7	36.2	3.9	0.27	43.0	34.8	2.9	0.34
Niacin (mg)	38.7	40.1	3.4	0.31	41.1	40.1	3.4	0.35	42.5	41.1	2.9	0.38
Vitamin E (mg)	35.3	38.7	5.8	0.23	34.3	37.7	4.8	0.21	39.1	36.2	3.4	0.29
Sodium (mg)	27.5	44.9	8.2	0.13	30.4	42.5	7.7	0.16	31.4	44.9	9.2	0.19
Calcium (mg)	38.7	43.0	5.3	0.32	37.2	35.3	5.3	0.23	41.6	39.6	4.8	0.34
Iron (mg)	39.1	44.4	2.9	0.36	42.0	38.7	1.9	0.36	42.5	40.6	1.9	0.39
Vitamin C (mg)	31.9	41.1	8.2	0.17	31.4	40.1	7.3	0.17	30.4	47.3	7.7	0.20
Cholesterol(mg)	39.1	38.2	4.4	0.30	38.2	38.7	5.8	0.27	38.2	38.7	2.9	0.30

a mean of FFQ1 and FFQ2.

### Reproducibility

For the food groups between FFQ1 and FFQ2, Spearman correlation coefficients ranged from 0.23 for pickled vegetable to 0.61 for fruit; Pearson correlation coefficients ranged from 0.27 for cooking oil to 0.64 for fruit; and the ICC ranged from 0.26 for cooking oil to 0.65 for fruit (Table 3). The proportion of subjects classified into one quartile (in the same and adjacent categories) by both FFQs ranged from 70% for vegetable to 87% for meat. Extreme misclassification into opposite quartiles was<7% with the exception of pickled vegetable. Weighted kappa values ranged from 0.16 for pickled vegetable and fresh vegetable to 0.50 for milk (Table 4).

For total energy and nutrient intakes between FFQ1and FFQ2, Spearman correlations ranged from 0.25 for sodium to 0.61 for protein; Pearson correlations ranged from 0.28 for carotene to 0.64 for cholesterol. The average ICC was 0.46 (0.28–0.62) (Table 3). The proportion of subjects classified into one quartile (in the same and adjacent categories) by both FFQs ranged from 73% for sodium to 86% for iron. Extreme misclassification into opposite quartiles was smaller than 7% except for carotene and sodium. The weighted kappa statistic showed fair to moderate conformity, ranging from 0.20 to 0.50, except for pickled vegetable and fresh vegetable that showed slight conformity (Table 4).

The average Pearson correlation coefficients between FFQ1 and FFQ2 in men ranged from 0.19 to 0.68, with an average of 0.43. In women, it ranged from 0.23 to 0.63, with an average of 0.43. The average ICCs were 0.42 in both genders and the average kappa values was 0.31 in men and 0.34 in women.

**Figure 2:**
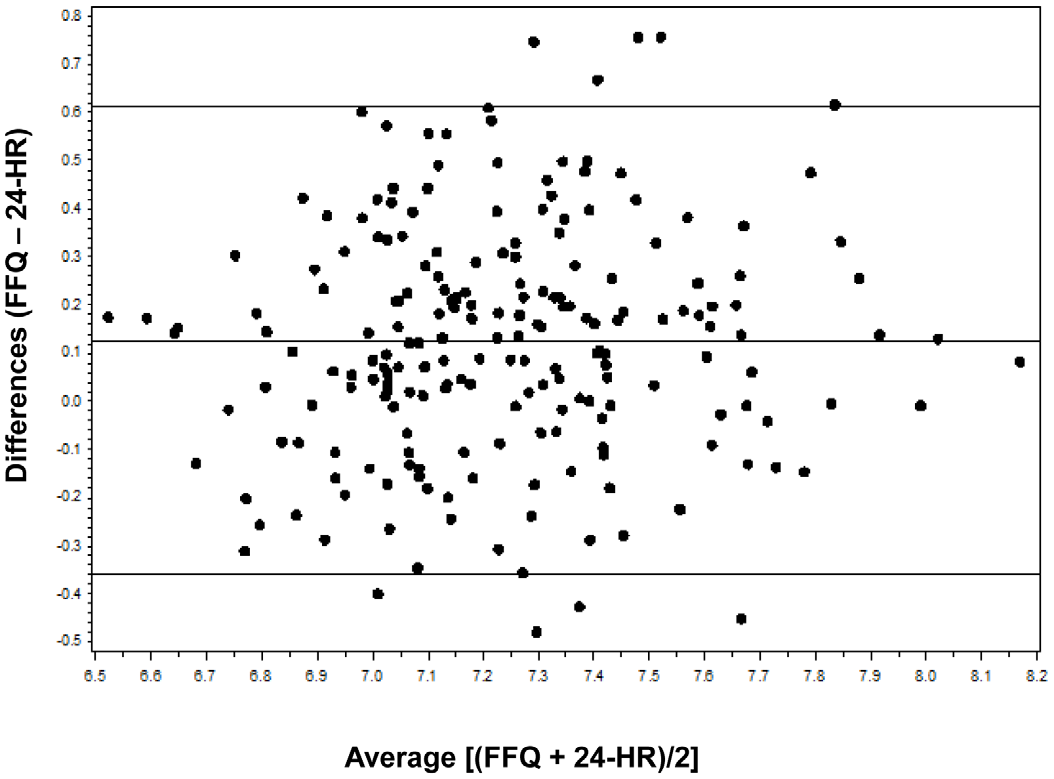
Bland Altman plot analysis of total energy intake. The Y axis is the difference between log-transformed data of total energy intake measured by FFQ (average of FFQ1 and FFQ2) and 24-HRs (average of the four 24-HRs). The × axis is the mean energy intake of the two methods. The central solid horizontal line represents the mean difference between the two methods, and the solid lines above and below it are ±2SDs.

**Figure 3:**
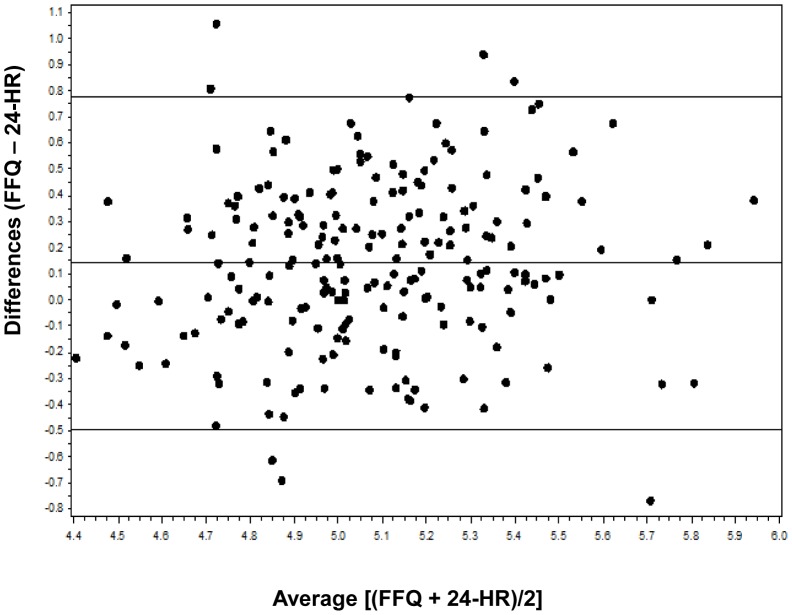
Bland Altman plot analysis of carbohydrate intake. The Y axis is the difference between log-transformed data of carbohydrate intake measured by FFQ (average of FFQ1 and FFQ2) and 24-HRs (average of the four 24-HRs). The × axis is the mean carbohydrate intake of the two methods. The central solid horizontal line indicates the mean difference between the two methods, and the solid lines above and below it indicate ±2SDs.

### Relative Validity

The crude, energy-adjusted, and de-attenuated Spearman and Pearson correlation coefficients of the FFQs (FFQ1, FFQ2, and averaged FFQ) and the mean of the four 24-HRs are presented in Table 5 and 6.

The crude Spearman correlation coefficients for food groups between FFQ2 and the 24-HRs ranged from 0.17 for pickled vegetables to 0.59 for fruit with an average of 0.41; the energy adjusted correlation coefficients ranged from 0.10 for pickled vegetable to 0.57 for cereal; and the de-attenuated coefficients ranged from 0.11 for pickled vegetable to 0.64 for fruit (Table 5). The crude Spearman correlation coefficients for nutrients and total energy between FFQ2 and the 24-HRs ranged from 0.20 for carotene to 0.58 for protein; the energy adjusted correlation coefficients ranged from 0.08 for iron to 0.54 for carbohydrate; and the de-attenuated coefficients ranged from 0.09 for iron to 0.56 for carbohydrate (Table 5).

The crude Pearson correlation coefficients for food groups between FFQ2 and the 24-HRs ranged from 0.14 for pickled vegetable to 0.65 for fruit; the energy adjusted coefficients ranged from 0.13 for pickled vegetable to 0.59 for cereal; and the de-attenuated coefficients ranged from 0.15 for pickled vegetable to 0.61 for cereal. The crude Pearson correlation coefficients for nutrients and energy between FFQ2 and the 24-HRs ranged from 0.21 for carotene to 0.61 for energy and protein; the energy adjusted coefficients ranged from 0.11 for vitamin A to 0.51 for carbohydrate; and the de-attenuated coefficients ranged from 0.12 for vitamin A to 0.53 for carbohydrate (Table 6).

The classification in quartiles (Table 7) yielded similar results for both FFQs with an average of more than 75% of the subjects classified into the same or adjacent quartiles by both methods. The proportion of subjects classified into one quartile (in the same/adjacent category) by FFQ2 and 24-HRs ranged from 67% for vitamin A to 83% for cereal and energy. Extreme misclassification of participants in opposite quartiles was<10% for all nutrients and food groups, with exception of pickled vegetable (10.6%), and snack and nut (11.1%) for FFQ2. The weighted kappa values for nutrients and food groups of the FFQs and the 24-HRs are also shown in Table 7. The values for FFQ2 and 24-HRs showed slight to fair conformity with all<0.40. With the mean of FFQ1 and FFQ2, the results showed a moderate conformity for cereal, meat, and energy (0.45, 0.46, and 0.44, respectively).

The average Spearman or Pearson correlation coefficients and kappa values between FFQ2 and 24-HRs were higher among men than women (0.35 vs. 0.32 for Spearman correlation coefficient; 0.37 vs. 0.34 for Pearson correlation coefficient; and 0.24 vs. 0.22 for kappa value).

Bland Altman plots demonstrated that the differences increased as the magnitude of the measurement increased, therefore the data was log-transformed and re-plotted (Figure 2 and Figure 3). The anti-log of the mean differences showed that FFQ overestimated nutrient intakes compared to 24-HR. For almost all food groups and nutrients, fewer than 10% of subjects were out of limits of agreement (LOA). But the anti-log of LOA indicated wide discrepancies between the two methods.

## Discussion

### Reproducibility

The median intakes for almost all nutrients obtained from FFQ2 were lower or equal to the values from FFQ1except for fat, vitamin E, cholesterol. This might be explained by the learning effect. Participants might estimate the amount more precisely after the previous surveys.

The correlation coefficients for reproducibility in our study are comparable to another validation study conducted in Shanghai in which the Spearman correlation coefficients range from 0.39 to 0.64 for food groups and 0.38 to 0.53 for nutrients, and the ICCs are 0.39 to 0.64 for food groups and 0.38 to 0.53 for nutrients [18]. Compared to other studies [19], [20], [21], the estimated correlation coefficients in the present study were slightly lower. In a study conducted in northern China, the ICCs between FFQ1 and FFQ2 are 0.40 to 0.80 for nutrients and food groups [21]. A possible explanation for the slightly lower correlations in our study was the long interval between FFQ1 and FFQ2. Various time intervals between FFQ1 and FFQ2, from 15 days to several years, have been reported in previous studies [22], [23]. If the two FFQs were administered closely, the correlations would be high, but overestimation might be resulted because subjects are more likely to remember and repeat their responses. In order to avoid the above error and decrease the variation of seasons, we performed FFQ1 and FFQ2 with an interval of nine months. Long interval may result in low correlation coefficients because differences in responses may reflect true changes in dietary habits as well as variation in response. Complication of the Chinese food preparation may be also responsible for the low correlation coefficients. In China, people usually mix several food items together, which makes it difficult to estimate the accurate amount of each food item.

The percentages of participants correctly classified into the same, adjacent, or extreme quartiles and the weighted kappa values are comparable to those reported by other validation studies [18], [21], [22]. In the Shanghai men’s study, the agreement rates for classifying nutrient and food group intakes into the same or adjacent categories are 73.8% to 91.8% [18]. In another study, the rates range from 70.8% to 92.9%, however, the weighted kappa values (range from 0.35 to 0.60) are higher than the values in the present study [21].

Some studies have reported the influence of gender on reproducibility of FFQ and the results are controversial [9], [24], [25], [26]. In the present study no difference was observed in the reproducibility between men and women, no matter Pearson correlation coefficient or ICC was used. The result is consistent with previous studies [25], [26].

### Relative Validity

In this study, nutrient intakes assessed by FFQ were higher than the intakes calculated by the average of the four 24-HRs. The mean differences showed in Bland-Altman plots were all positive. These findings was consistent with the results reported in other studies [11], [20], [27], [28]. A possible explanation is that the subjects might recall some food items more than once when they ate the foods in a mixed dish.

Masson et al. [29] suggested that a correlation coefficient above 0.5 is desirable for validation studies. In the present study, the correlation coefficients for some nutrients did not reach that threshold. Other studies conducted in Chinese population have reported correlation coefficients ranging from 0.25 to 0.72 [18], [21], [30], [31]. Kim et al. examined the validity of nutrient assessment using an 80-item FFQ and obtained a range of energy-adjusted correlation coefficients against 24-HRs from 0.08 for zinc to 0.34 for calcium [32]. The types of food items included in the FFQs and the repeated number of 24-HR may influence the validity of the FFQ. The present FFQ was developed based mainly on individual food items, not on prepared dishes. But there is no definitive evidence suggesting that a dish-based FFQ is more precise in assessing dietary intakes. In our study, there were four 24-HRs, one for each season. If the frequency of consumption is low and the within-person variability is too high, the correlation coefficients can be attenuated [33], because the probability of assessing rarely consumed foods on the four 24-HRs is low. Data from the four 24-HRs indicated that vegetables consumed in different seasons varied significantly. People consumed more seasonal vegetables. For example, in winter season people consumed more vegetables that can still plant or could be stored in winter such as cabbage, carrot, and radish. For fruits, there are few fruits to eat; therefore, limited fruits were consumed for participants. Difficulties in portion size estimations may also bias the true validity of the FFQ. Energy-adjustment led to the validity correlation decrease for almost all food groups and nutrients, which may be due to high between-person variation in the intakes of food groups and nutrients in our study subjects.

Despite some differences in estimation of both nutrients and food groups, we obtained a reasonable agreement in classification. More than 67% of the subjects were classified into the same or adjacent quartile for food group and nutrient intakes by both methods, which is consistent with other studies [18], [21], [27], [30], [31], [34]. A moderate agreement (weighted kappa>0.40) was observed for cereal, meat, and energy. An acceptable agreement (kappa 0.20 to 0.39) was obtained for most nutrients and food groups.

Log-transformation was performed in Bland-Altman analysis because the differences increased as the magnitude of the measurement increased. Bland-Altman plots demonstrated that the FFQ overestimated intakes for most food groups and all nutrients, similar to the results of Watson’s study [11]. Although the LOA was wide, the mean differences of nutrient intakes were around zero indicating that the FFQ is not suitable for estimating absolute intakes, but is appropriate for ranking intakes.

In the present study, the relative validity was higher in men than in women. The result was consistent with another study conducted in Iran in which the mean energy-adjusted and deattenuated correlation coefficients were 0.53 and 0.39 in men and women, respectively [26]. They thought the differences may be due to the same portion sizes being used for men and women. But we did not use unified portion sizes. A possible explanation in the present study is that women are more concerned about their body weight and tend not to answer the true amount of foods consumed.

There are some limitations in the design of FFQ and the implementation of validation study. First, beverage was not included except for alcohol, which might influence the energy intake and lead to decreased validity. However, the beverage consumption is very limited in this population. Second, during the third recall, one interviewer was on sick leave and the subjects who should be interviewed by the interviewer were instead interviewed by others. Some participants were not interviewed during the second FFQ and instead the data were obtained from the people living together with the participants. We think that this might bring some influence on the reproducibility and validity of the FFQ. Third, the data would be more representative if 24-HRs were collected monthly, instead of every quarter. Last, we did not analyze the independent influence of age, BMI, and education level on the reproducibility and validity, but the correlation coefficients adjusted for these factors did not change materially.

### Conclusion

This study evaluated the validity and reproducibility of an 86-item FFQ developed specifically for investigation of the relationship between dietary factors and chronic diseases in Taizhou Longitudinal Study. The results in the present study demonstrated that the ability to rank subjects according to the nutrient intakes obtained from the FFQ was reasonably acceptable for most nutrients and foods in the study population.

## Supporting Information

Table S1 Correlation coefficients of FFQ1 and FFQ2 for men and women.
(DOC)

Table S2 Spearman correlation coefficients of food groups and nutrients estimated from FFQs and 24-HRs for men.
(DOC)

Table S3 Spearman correlation coefficients of food groups and nutrients estimated from FFQs and 24-HRs for women.
(DOC)
