# Supplementary material for: Reproducibility and Relative Validity of a Food Frequency Questionnaire Developed for Adults in Taizhou, China
Source: PLoS One. 2012 Nov 6;7(11):e48341. doi: 10.1371/journal.pone.0048341 (PMC3491058; doi:10.1371/journal.pone.0048341)
Supplement: Table S1 — Correlation coefficients of FFQ1 and FFQ2 for men and women. (DOC) [file pone.0048341.s001.doc]

Table S1. Correlation coefficients of FFQ1 and FFQ2 for men and women

|  |  |  | | Men | |  | |  |  | | Women | |  | |
| --- | --- | --- | --- | --- | --- | --- | --- | --- | --- | --- | --- | --- | --- | --- |
| variables |  | Spearman | | Pearson* | | ICC* | |  | Spearman | | Pearson* | | ICC* | |
|  |  | Crude | Energy-  adjusted | Crude | Energy-  adjusted | Crude | Energy-  adjusted |  | Crude | Energy-  adjusted | Crude | Energy-  adjusted | Crude | Energy-  adjusted |
| Food groups |  |  |  |  |  |  |  |  |  |  |  |  |  |  |
| Cereal (g) |  | 0.38 | 0.45 | 0.36 | 0.44 | 0.31 | 0.41 |  | 0.56 | 0.52 | 0.52 | 0.55 | 0.44 | 0.45 |
| Pickled vegetable (g) |  | 0.32 | 0.27 | 0.31 | 0.22 | 0.34 | 0.29 |  | 0.14 | 0.13 | 0.26 | 0.13 | 0.25 | 0.26 |
| Egg (g) |  | 0.36 | 0.55 | 0.30 | 0.45 | 0.34 | 0.29 |  | 0.51 | 0.43 | 0.49 | 0.50 | 0.51 | 0.50 |
| Meat (g) |  | 0.44 | 0.37 | 0.48 | 0.34 | 0.48 | 0.30 |  | 0.54 | 0.41 | 0.52 | 0.42 | 0.51 | 0.48 |
| Fish and  shellfish (g) |  | 0.69 | 0.40 | 0.63 | 0.54 | 0.63 | 0.52 |  | 0.41 | 0.24 | 0.41 | 0.24 | 0.41 | 0.37 |
| Milk (g) |  | 0.57 | 0.40 | 0.58 | 0.40 | 0.56 | 0.34 |  | 0.49 | 0.40 | 0.40 | 0.48 | 0.49 | 0.51 |
| Snack and nut (g) |  | 0.47 | 0.41 | 0.52 | 0.65 | 0.48 | 0.41 |  | 0.53 | 0.56 | 0.54 | 0.39 | 0.51 | 0.44 |
| Bean (g) |  | 0.35 | 0.34 | 0.26 | 0.31 | 0.27 | 0.27 |  | 0.66 | 0.59 | 0.62 | 0.57 | 0.61 | 0.54 |
| Vegetable (g) |  | 0.14 | -0.02 | 0.19 | 0.05 | 0.18 | 0.06 |  | 0.32 | 0.22 | 0.34 | 0.20 | 0.33 | 0.26 |
| Fruit (g) |  | 0.59 | 0.43 | 0..68 | 0.43 | 0.67 | 0.61 |  | 0.61 | 0.54 | 0.62 | 0.51 | 0.64 | 0.62 |
| Cooking oil (g) |  | 0.36 | 0.30 | 0.41 | 0.31 | 0.28 | 0.29 |  | 0.34 | 0.27 | 0.25 | 0.27 | 0.25 | 0.31 |
| Nutrients |  |  |  |  |  |  |  |  |  |  |  |  |  |  |
| Energy (Kcal) |  | 0.57 | − | 0.54 | − | 0.53 | − |  | 0.52 | − | 0.48 | − | 0.45 | − |
| Protein (g) |  | 0.57 | 0.43 | 0.55 | 0.52 | 0.54 | 0.49 |  | 0.55 | 0.38 | 0.52 | 0.38 | 0.50 | 0.39 |
| Fat (g) |  | 0.46 | 0.28 | 0.45 | 0.28 | 0.45 | 0.29 |  | 0.34 | 0.26 | 0.29 | 0.28 | 0.29 | 0.29 |
| Fiber (g) |  | 0.48 | 0.38 | 0.49 | 0.38 | 0.49 | 0.34 |  | 0.51 | 0.34 | 0.49 | 0.34 | 0.48 | 0.34 |
| Carbohydrate (g) |  | 0.42 | 0.57 | 0.38 | 0.54 | 0.34 | 0.46 |  | 0.55 | 0.30 | 0.51 | 0.33 | 0.44 | 0.17 |
| Vitamin A (μg) |  | 0.33 | 0.12 | 0.36 | 0.16 | 0.36 | 0.20 |  | 0.36 | 0.19 | 0.32 | 0.17 | 0.31 | 0.24 |
| Carotene (μg) |  | 0.27 | 0.13 | 0.29 | 0.16 | 0.28 | 0.23 |  | 0.28 | 0.16 | 0.28 | 0.16 | 0.27 | 0.22 |
| Thiamin (mg) |  | 0.51 | 0.52 | 0.50 | 0.47 | 0.49 | 0.39 |  | 0.58 | 0.25 | 0.55 | 0.34 | 0.51 | 0.28 |
| Riboflavin (mg) |  | 0.56 | 0.40 | 0.55 | 0.32 | 0.55 | 0.29 |  | 0.42 | 0.30 | 0.43 | 0.28 | 0.41 | 0.29 |
| Niacin (mg) |  | 0.44 | 0.44 | 0.45 | 0.48 | 0.45 | 0.43 |  | 0.51 | 0.34 | 0.43 | 0.30 | 0.42 | 0.22 |
| Vitamin E (mg) |  | 0.46 | 0.35 | 0.41 | 0.36 | 0.39 | 0.28 |  | 0.41 | 0.24 | 0.33 | 0.26 | 0.33 | 0.28 |
| Sodium (mg) |  | 0.30 | 0.15 | 0.33 | 0.15 | 0.33 | 0.20 |  | 0.15 | 0.11 | 0.23 | 0.11 | 0.21 | 0.21 |
| Calcium (mg) |  | 0.39 | -0.09 | 0.42 | 0.06 | 0.42 | 0.09 |  | 0.39 | 0.27 | 0.40 | 0.29 | 0.38 | 0.33 |
| Iron (mg) |  | 0.50 | 0.16 | 0.44 | 0.22 | 0.44 | 0.25 |  | 0.55 | 0.41 | 0.51 | 0.32 | 0.48 | 0.28 |
| Vitamin C (mg) |  | 0.32 | 0.05 | 0.30 | 0.10 | 0.29 | 0.16 |  | 0.37 | 0.23 | 0.37 | 0.20 | 0.35 | 0.26 |
| Cholesterol (mg) |  | 0.52 | 0.64 | 0.56 | 0.38 | 0.56 | 0.43 |  | 0.54 | 0.53 | 0.63 | 0.57 | 0.60 | 0.62 |

*data were log-transformed
