# Supplementary material for: Reproducibility and Relative Validity of a Food Frequency Questionnaire Developed for Adults in Taizhou, China
Source: PLoS One. 2012 Nov 6;7(11):e48341. doi: 10.1371/journal.pone.0048341 (PMC3491058; doi:10.1371/journal.pone.0048341)
Supplement: Table S3 — Spearman correlation coefficients of food groups and nutrients estimated from FFQs and 24-HRs for women. (DOC) [file pone.0048341.s003.doc]

Table S3. Spearman correlation coefficients of food groups and nutrients estimated from FFQs and 24-HRs for women

| variables |  | FFQ1 *vs.* 24-HRs | | |  | FFQ2 *vs.* 24-HRs | | |  | Mean * *vs.* 24-HRs | | |
| --- | --- | --- | --- | --- | --- | --- | --- | --- | --- | --- | --- | --- |
|  |  | R^a^ | R^b^ | R^c^ |  | R^a^ | R^b^ | R^c^ |  | R^a^ | R^b^ | R^c^ |
| Food groups |  |  |  |  |  |  |  |  |  |  |  |  |
| Cereal (g) |  | 0.61 | 0.55 | 0.56 |  | 0.53 | 0.60 | 0.62 |  | 0.66 | 0.67 | 0.69 |
| Pickled vegetable (g) |  | 0.05 | 0.08 | 0.09 |  | 0.19 | 0.20 | 0.23 |  | 0.20 | 0.17 | 0.19 |
| Egg (g) |  | 0.17 | 0.35 | 0.38 |  | 0.09 | 0.14 | 0.15 |  | 0.17 | 0.29 | 0.32 |
| Meat (g) |  | 0.40 | 0.28 | 0.32 |  | 0.40 | 0.31 | 0.35 |  | 0.49 | 0.35 | 0.39 |
| Fish and shellfish (g) |  | 0.27 | 0.30 | 0.34 |  | 0.39 | 0.35 | 0.40 |  | 0.39 | 0.39 | 0.45 |
| Milk (g) |  | 0.27 | 0.39 | 0.43 |  | 0.26 | 0.43 | 0.48 |  | 0.29 | 0.47 | 0.52 |
| Snack and nut (g) |  | 0.47 | 0.56 | 0.60 |  | 0.35 | 0.67 | 0.71 |  | 0.48 | 0.67 | 0.71 |
| Bean (g) |  | 0.50 | 0.43 | 0.46 |  | 0.46 | 0.42 | 0.45 |  | 0.51 | 0.47 | 0.51 |
| Vegetable (g) |  | 0.28 | 0.22 | 0.24 |  | 0.28 | 0.21 | 0.23 |  | 0.34 | 0.27 | 0.30 |
| Fruit (g) |  | 0.56 | 0.47 | 0.58 |  | 0.35 | 0.42 | 0.52 |  | 0.62 | 0.53 | 0.66 |
| Cooking oil (g) |  | 0.10 | 0.11 | 0.13 |  | 0.25 | 0.18 | 0.20 |  | 0.23 | 0.20 | 0.23 |
| Nutrients |  |  |  |  |  |  |  |  |  |  |  |  |
| Energy (Kcal) |  | 0.41 | − | − |  | 0.32 | − | − |  | 0.42 | − | − |
| Protein (g) |  | 0.45 | 0.37 | 0.40 |  | 0.44 | 0.32 | 0.34 |  | 0.49 | 0.41 | 0.44 |
| Fat (g) |  | 0.33 | 0.34 | 0.36 |  | 0.42 | 0.33 | 0.35 |  | 0.46 | 0.43 | 0.46 |
| Fiber (g) |  | 0.19 | -0.11 | -0.12 |  | 0.24 | 0.10 | 0.11 |  | 0.25 | -0.01 | -0.01 |
| Carbohydrate (g) |  | 0.39 | 0.43 | 0.45 |  | 0.31 | 0.41 | 0.43 |  | 0.41 | 0.53 | 0.55 |
| Vitamin A (μg) |  | 0.29 | 0.21 | 0.23 |  | 0.26 | 0.25 | 0.28 |  | 0.36 | 0.29 | 0.32 |
| Carotene (μg) |  | 0.28 | 0.13 | 0.15 |  | 0.26 | 0.21 | 0.24 |  | 0.38 | 0.21 | 0.24 |
| Thiamin (mg) |  | 0.26 | 0.09 | 0.10 |  | 0.22 | 0.16 | 0.18 |  | 0.27 | 0.17 | 0.19 |
| Riboflavin (mg) |  | 0.34 | 0.10 | 0.11 |  | 0.30 | 0.21 | 0.23 |  | 0.38 | 0.18 | 0.20 |
| Niacin (mg) |  | 0.35 | 0.15 | 0.17 |  | 0.26 | 0.11 | 0.13 |  | 0.35 | 0.14 | 0.16 |
| Vitamin E (mg) |  | 0.32 | 0.32 | 0.34 |  | 0.39 | 0.25 | 0.26 |  | 0.44 | 0.37 | 0.39 |
| Sodium (mg) |  | 0.15 | 0.11 | 0.12 |  | 0.27 | 0.22 | 0.24 |  | 0.28 | 0.19 | 0.21 |
| Calcium (mg) |  | 0.28 | 0.21 | 0.23 |  | 0.27 | 0.22 | 0.24 |  | 0.34 | 0.26 | 0.28 |
| Iron (mg) |  | 0.41 | 0.33 | 0.36 |  | 0.39 | 0.21 | 0.23 |  | 0.45 | 0.32 | 0.35 |
| Vitamin C (mg) |  | 0.33 | 0.24 | 0.27 |  | 0.27 | 0.18 | 0.20 |  | 0.36 | 0.27 | 0.30 |
| Cholesterol (mg) |  | 0.37 | 0.36 | 0.39 |  | 0.39 | 0.38 | 0.41 |  | 0.44 | 0.43 | 0.47 |

^a^ crude Spearman correlation coefficients

^b^ energy-adjusted Spearman correlation coefficients

^c^ energy-adjusted and de-attenuated Spearman correlation coefficients
